# Case of Supracristal Ventricular Septal Defect and Aortic Regurgitation Detected by Cardiac Auscultation but Missed by Diagnostic Imaging

**DOI:** 10.7759/cureus.13502

**Published:** 2021-02-23

**Authors:** Karthik Seetharam, Roman Pachulski

**Affiliations:** 1 Cardiology, St. John's Episcopal Hospital, New York, USA

**Keywords:** cardiac auscultation, imaging, murmurs

## Abstract

Imaging technology has diminished the reliance on cardiac auscultation as a definitive diagnostic tool. However, it retains relevance in its immediacy, minimal preparation, and power source independence. We present a case of clinically detected continuous murmur raising specific diagnostic possibilities not accounted for advanced imaging. Further testing revealed a large supracristal ventricular septal defect (VSD) and aortic regurgitation (AR), allowing the surgeon to anticipate combined septal and valvular surgery. This report highlights the value of cardiac auscultation as a guide and validation for imaging. The absence of lesions on imaging is not proof of lesion absence.

## Introduction

Cardiac auscultation was once heralded as the cornerstone of physical examination is slowly being replaced by rapidly emerging technology [1,2]. With the advent of handheld ultrasound, auscultation and physical examination have been de-emphasized [3]. Excessive reliance on developing technology combined with diminished clinical proficiency may prove misleading in clinical practice [2]. We describe a case of hemodynamically significant continuous murmur readily detectable during auscultation. A continuous murmur is defined as a constant intensity murmur throughout systole that extends beyond S2 but not necessarily through all diastole. Although not common it raises a specific list of possible diagnoses including post-semilunar valve to right heart shunts (patent ductus arteriosus, aortopulmonary window, sinus of Valsalva aneurysm, an anomalous left coronary artery from the pulmonary artery), and mimic lesions including supracristal ventricular septal defect (VSD )with aortic regurgitation (AR), pulmonary artery branch stenosis and coarctation. Imaging was interpreted as documenting only aortic regurgitation, which can often be associated with a short systolic flow murmur, it will not persist through all of the systole. Despite a recommendation to proceed with valvular surgery, further imaging was undertaken to account for initial auscultatory findings and revealed a large supracristal VSD. This allowed the surgeon to plan combined septal and valvular surgery.

## Case presentation

An 18-year-old asthenic Nigerian male was referred for murmur evaluation detected on pre-employment physical examination. He noted New York Heart Association (NYHA) 2 exertional intolerant (>10 minutes of physical activity) but denied a history of resting dyspnea, syncope, or chest pain. On physical examination, a four of six constant intensity murmur was identified from S1 and extended beyond S2 midway into diastole. In addition, the murmur was loudest at the second left intercostal space radiating to the mid-scapula posteriorly. A palpable left lower sternal border systolic thrill was appreciated and the cardiac apex was displaced to the 6th intercostal space at the midaxillary line. Electrocardiogram (EKG) revealed normal sinus rhythm with 80 beats per minute with V3 R and S wave of equal amplitude whose sum exceeded 45mm (Katz-Wachtel pattern) suggestive of biventricular enlargement. Furthermore, V5 and V6 had deep Q waves and upright T waves consistent with volume overload of the left ventricle. Chest x-ray (CXR) confirmed cardiomegaly with enlarged right atrium (RA), pulmonary artery (PA), right ventricle. The initial transthoracic echocardiogram (TTE) documented severe left ventricle (LV) dilatation, ejection fraction (EF) of 45%, mild aortic root dilatation, trace aortic regurgitation, trileaflet valve, and PA dilatation. A shunt was not visualized. Subsequently, a transesophageal echocardiogram (TEE) was suggested. However, the patient did not attend to TEE or follow-up in six months. 

**Figure 1:**
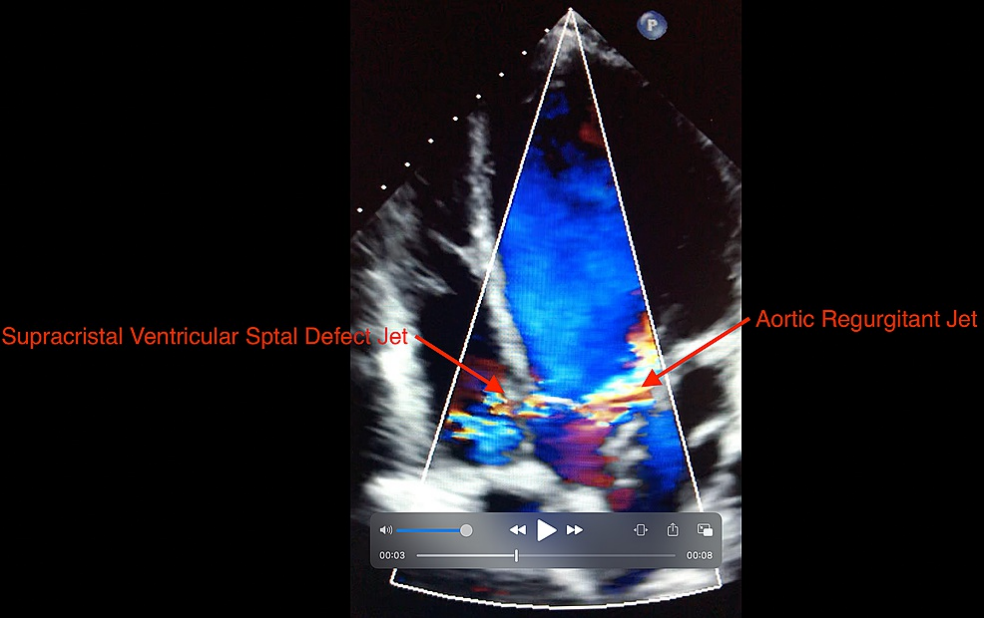
TEE showing supracristal VSD and AR jet TEE- transesophageal echocardiogram; VSD- ventricular septal defect; AR- aortic regurgitation

**Figure 2:**
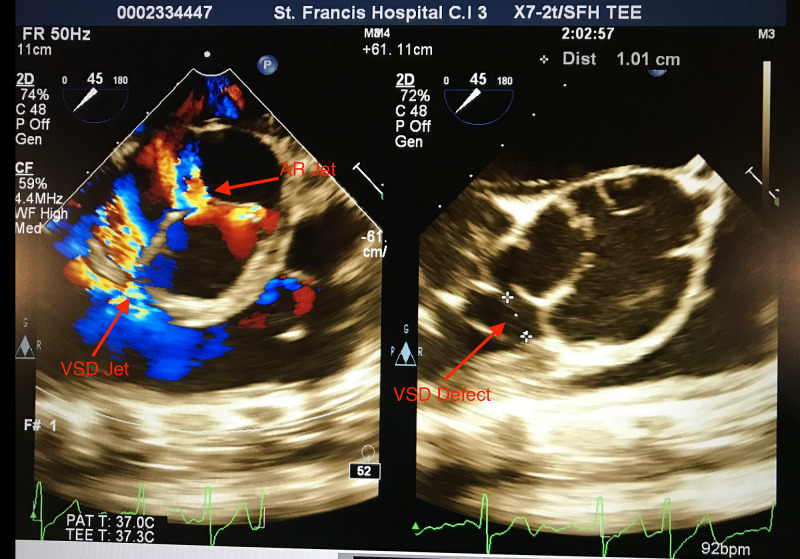
TEE showing the location of VSD jet and AR jet TEE- transesophageal echocardiogram; VSD- ventricular septal defect; AR- aortic regurgitation

Upon his return, pediatric cardiology consultation was requested and MRI was obtained. The MRI was reported as documenting severe AR with a trileaflet valve and no evidence of aortopathy or coronary pathology. A Qp/Qs (pulmonary flow/systemic flow) was reported but dismissed. Valvular surgery was recommended. The discordance between auscultatory findings and MRI prompted TEE imaging. It revealed a supracristal VSD (Figure 1) with severe AR (Video 1). The location of such a VSD just under the aortic valve, not infrequently draw the adjacent right coronary cusp into the defect by a Venturi effect of the left ventricular to right ventricular shunt across the interventricular septum (Figure 2; Video 2). In addition, there was irrevocable damage to the right and non-coronary cusps and partially obstructing the VSD.

**Video 1:** TEE showing supracristal VSD jet TEE- transesophageal echocardiogram; VSD- ventricular septal defect

**Video 2:** TEE showing VSD jet and AR jet TEE- transesophageal echocardiogram; VSD- ventricular septal defect; AR- aortic regurgitation

The VSD was patched with the pericardium, a #25 mm mechanical St Jude's prosthesis was placed in the aortic position successfully. Postoperative TEE revealed no discernable shunt or paravalvular AR. Furthermore, the blood gas samples showed no SaO2 step up. The patient recovered well and was discharged home with warfarin. Follow-up TTE at six months showed a reduction in LV size and an increase in left ventricle ejection fraction (LVEF) to 55%. 

## Discussion

In order to achieve high interpretative precision with cardiac auscultation frequent repetition with an expert review is necessary [1]. Cardiology fellowships are increasingly dominated by highly technical imaging techniques minimizing time devoted to auscultation [1]. Advanced, particularly non-invasive imaging techniques add immeasurably to diagnostic accuracy prior to procedural intervention. Many imaging findings can be consistent with a plurality of diagnoses that must be interpreted in light of clinical evaluation. 

TTE differed from TEE with regard to AR severity, emphasizing the potential for under-detection by planar imaging. This is a consequence of technical image acquisition or window availability. TEE verified interventricular septal discontinuity but minimized the shunt volume. This is commonly seen with supracristal VSD as the right coronary cusp prolapses (due to the venturi effect of the adjacent left-to-right shunt) across the defect partially occluding it. This could have been demonstrated with right and left heart catheterization with sequential oxygen saturation run. However modern management seeks to minimize preoperative invasive evaluation unless percutaneous remediation is contemplated. At operation, the VSD was measured at 20 mm diameter. The MRI measured parameters listed a Qp/Qs= 1.34:1 that is consistent with a significant (> 1.2), though not severe shunt. Nonetheless, it was formally reported as excluding the presence of a shunt. 

In our case, the clinical examination and CXR clearly pointed towards a possible shunt [3]. Isolated AR cannot produce the above discrepancy between pulmonic and systemic flow and cannot cause right heart chamber distortions. Many imaging modalities are planar raising the possibility of technical limitations. However, in this case, even MRI, an ostensibly three-dimensional imaging technique did not yield the correct diagnosis. Most importantly, the absence of a finding is not proof of its non-existence. When imaging is discordant with clinical evaluation, repeat alternate imaging is warranted. Although imaging quality is constantly improving, this does not diminish the immediacy and relevance of auscultatory findings. It can differ significantly even from ultrasound imaging [3].

Many institutions are allocating fewer resources for proper teaching and development of these vital skills [1]. Previous studies have documented a downwards trend in clinical proficiency in American and European countries with nearly 25% accuracy in cardiac auscultation among medicine residents [4,5]. This is an alarming finding. Technology should not replace, but rather supplement auscultation. Technology may provide a solution in the form of prerecorded auditory databases of heart sounds to facilitate repeat exposure expediting improved proficiency [2]. Cardiac auscultation still retains immediate clinical relevance at the bedside and should be the primary step in clinical evaluation before the application of various imaging tests. 

## Conclusions

Over-reliance on advanced imaging in a clinical vacuum of diminished or absent auscultation can lead to errors in cardiovascular diagnosis. The auscultatory findings can serve as a vital and necessary first step in management. We report such a significant auscultatory finding whose explanation was under-detected by TTE, TEE, and MRI. Identifying the clinical imaging discordance revealed the diagnosis and facilitated surgical intervention. 
